# Guanine Crystallization by Particle Attachment

**DOI:** 10.1021/jacs.5c04543

**Published:** 2025-05-23

**Authors:** Shashanka S. Indri, Florian M. Dietrich, Avital Wagner, Michal Hartstein, Einat Nativ-Roth, Mariela J. Pavan, Leeor Kronik, Matteo Salvalaglio, Benjamin A. Palmer

**Affiliations:** † Department of Chemistry, Ben-Gurion University of the Negev, Be’er Sheba 8410501, Israel; ‡ Department of Chemical Engineering, 4919 University College London, London WC1E 7JE, United Kingdom; § Department of Molecular Chemistry and Materials Science, 34976 Weizmann Institute of Science, Rehovoth 7610001, Israel; ∥ Ilse Katz Institute for Nanoscale Science and Technology, 26732 Ben-Gurion University of the Negev, Be’er Sheba 8410501, Israel

## Abstract

Understanding how crystals nucleate is a key goal in materials, biomineralization, and chemistry. Many inorganic materials are known to crystallize “nonclassically” by particle attachment. However, a molecular-level understanding of small molecule crystallization is hampered by the complexity and time scales of nucleation events, which are often too large to simulate and too small to observe. Here, by combining unbiased molecular dynamics simulations and *in situ* experiments, we uncover this nucleation “blind spot” to elucidate the nonclassical crystallization mechanism of the nucleobase, guanine. The multi-step nucleation process begins with stacked guanine clusters, whose H-bonding and π-stacking arrangement progressively orders as they attach into nanoscopic fibers (observed by simulation and electron microscopy), partially ordered bundles, and finally, 3D periodic crystals. This work provides a foundation for understanding how organisms exquisitely control the formation of guanine and other molecular crystals, which are used ubiquitously in biology as optical and nitrogen-storage materials.

## Graphical abstract

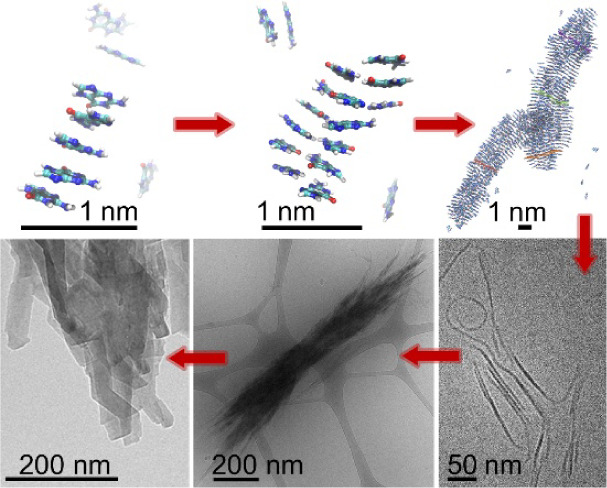

## Introduction

Crystallization is a ubiquitous phenomenon underpinning the formation of geological, synthetic, and biological materials. Classic crystallization models^1,2^ assume that a critical nucleus emerges from solution upon a sharp discontinuity in density and structure and then grows by monomer attachment. However, many inorganic materials, biominerals, and proteins form “nonclassically” ^3–5^ by a multi-step ordering of metastable states (e.g., liquid droplets,^6^ clusters,^7^ amorphous particles,^8,9^ and nanocrystals^10,11^ ), which often evolve into the final crystal by *particle attachment*.^12–14^ In contrast, molecular-level mechanisms of the complex nucleation pathways of small organic molecules remain elusive.^15^ A key problem is the difficulty of combining molecular information from simulation^16–18^ with direct observation of electron-diffuse molecules.^19^

Recently, there has also been a surge of interest in “organic biocrystallization” – the formation of molecular crystals by organisms.^20,21^ Guanine is the most widespread of these biocrystals – being used as an optical material in animals^22–24^ and for nitrogen storage in microalgae.^25^ Initially, guanine crystals were believed to adopt the α-polymorph (*P*2_1_/*c*) – the only known form at the time. However, subsequent studies using powder X-ray diffraction (PXRD) and density functional theory (DFT) revealed that biogenic guanine crystals adopt the kinetically accessible β-polymorph (*P*2_1_/*b*).^26^ Later, the use of three-dimensional electron diffraction (3D ED) enabled the determination of a more accurate β*-*guanine crystal structure (*P*2_1_/*c*), including the precise locations of the hydrogen atoms.^27^ Both polymorphs are comprised of π-stacked, H-bonded layers (Figure 1) and share identical H-bonding connectivity within these layers. The only difference lies in the relative offset of adjacent layers parallel to the *bc* plane, and they are therefore expected to have similar morphological and optical properties. Guanine crystals possess an extremely high refractive index within the H-bonded (100) plane (*n* = 1.83). Organisms exquisitely control the growth of these crystals by small molecule^28–30^ and macromolecular additives^31,32^ to produce plate-like crystals preferentially expressing high refractive index facets. However, though the self-assembly of guanine derivatives (e.g., guanosine,^33^ G-quadruplexes,^34^ FMOC-guanine^35^) have been widely explored,^36^ little is understood about the assembly and nucleation of the pure nucleobase itself.

**Figure 1:**
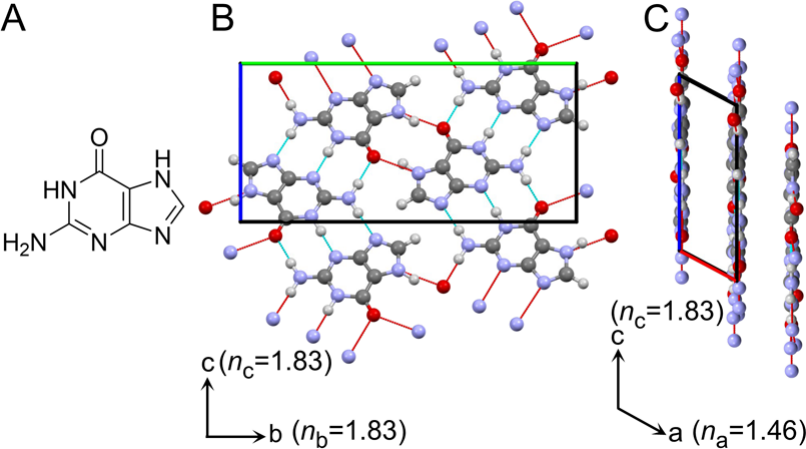
Structural properties of guanine. (A) Molecular structure of guanine. The β-guanine crystal structure viewed (B) perpendicular to the H-bonded (100) plane (refractive index in *bc* plane = 1.83) and (C) parallel to the π-stacking layers. Refractive index along *a* = 1.46.

Here, using *in situ* experiments, electronic structure calculations, and unbiased MD simulations, we show how guanine crystallizes by particle attachment of π-stacked molecular assemblies. The H-bonding and π-stacking arrangement of these assemblies gradually converges to that of the β-guanine crystal structure through the relaxation of structural defects. This fundamental understanding of crystallization is essential for rationalizing how organisms control guanine crystal morphology, for example, in their preferential expression of the highly reflective but hydrophobic (100) crystal face. We anticipate that similar nonclassical crystallization pathways, mediated by particle attachment, underlie the crystallization of many planar aromatic molecules, including other purines and pteridines, utilized in optically functional biogenic crystals.^37–40^

## Results and Discussion

### Morphological Evolution of Guanine during Self-Assembly and Nucleation

To investigate guanine crystallization, guanine (6.6 mM) was dissolved in aqueous solutions at pH 13.00 (Figure S1), and crystallization was induced by gradually lowering the pH to 11.35 (supersaturation, *S* ∼ 1.20).^41^ Decreasing the pH shifts the equilibrium from the soluble deprotonated form of guanine towards the insoluble neutral form (Figure S2), which increases the supersaturation and the rate of nucleation.^42,43^ At various time points after pH 11.35 had been reached, aliquots of the crystallization solution were collected from the middle of the container to ensure uniform sampling and subsequently imaged by cryogenic transmission electron microscopy (cryo-TEM). Initially (*t* = 30 s), flexible 1D fibers of varied lengths (114.25 ± 56.74 nm), but conserved widths (4.75 ± 0.56 nm), are observed in solution (Figures 2A, S3). As crystallization proceeds (*t* = 10 min), the fibers elongate (190 ± 107.6 nm, Figure S3) and subsequently thicken through fusion (Figure 2B, blue asterisks), with their widths remaining invariant during elongation and increasing only upon the merging of two or more fibers. Co-oriented assemblies of straightened fibers then form^5,19^ (Figures 2C inset and S4), with each fiber being separated by a well-defined aqueous layer (2.67 ± 0.38 nm). After 35 min, these fibers coalesce through a “nearly oriented attachment” process^12^ to form fibrous bundles, several micrometers long and hundreds of nanometers wide. These bundles exhibit intense {100} reflections (*d*-spacing = 3.2 Å), corresponding to the π-stacking distance between guanine planes (Figure 2D). The {100} reflections are oriented parallel to the long axis of the bundles, demonstrating that the guanine molecules are π-stacked along the long fiber axis. The large azimuthal spread (20°) of the {100} reflections indicates a high degree of orientational disorder in the π-stacking (Figure 2D, right inset). The absence of other diffraction peaks indicates a 1D ordered array of π-stacked guanine fibrils with no long-range ordering in the H-bonding plane. At this stage, some bundles also exhibit faceted regions (Figure 2D, left inset). As crystallization progresses (*t* = 65 min), the bundles develop a predominantly faceted character (Figure 2E, yellow arrow), displaying a “chevron” morphology – characteristic of the (100) face twinning of β-guanine crystals (Figure 2D).^44^ However, fibrous features are still present at the periphery (Figure 2E, blue arrow), indicating a process of continuous molecular rearrangement. The final precipitate (*t* = 120 min) consisted of prismatic crystals of β-guanine (Figures 2F and S5).

**Figure 2:**
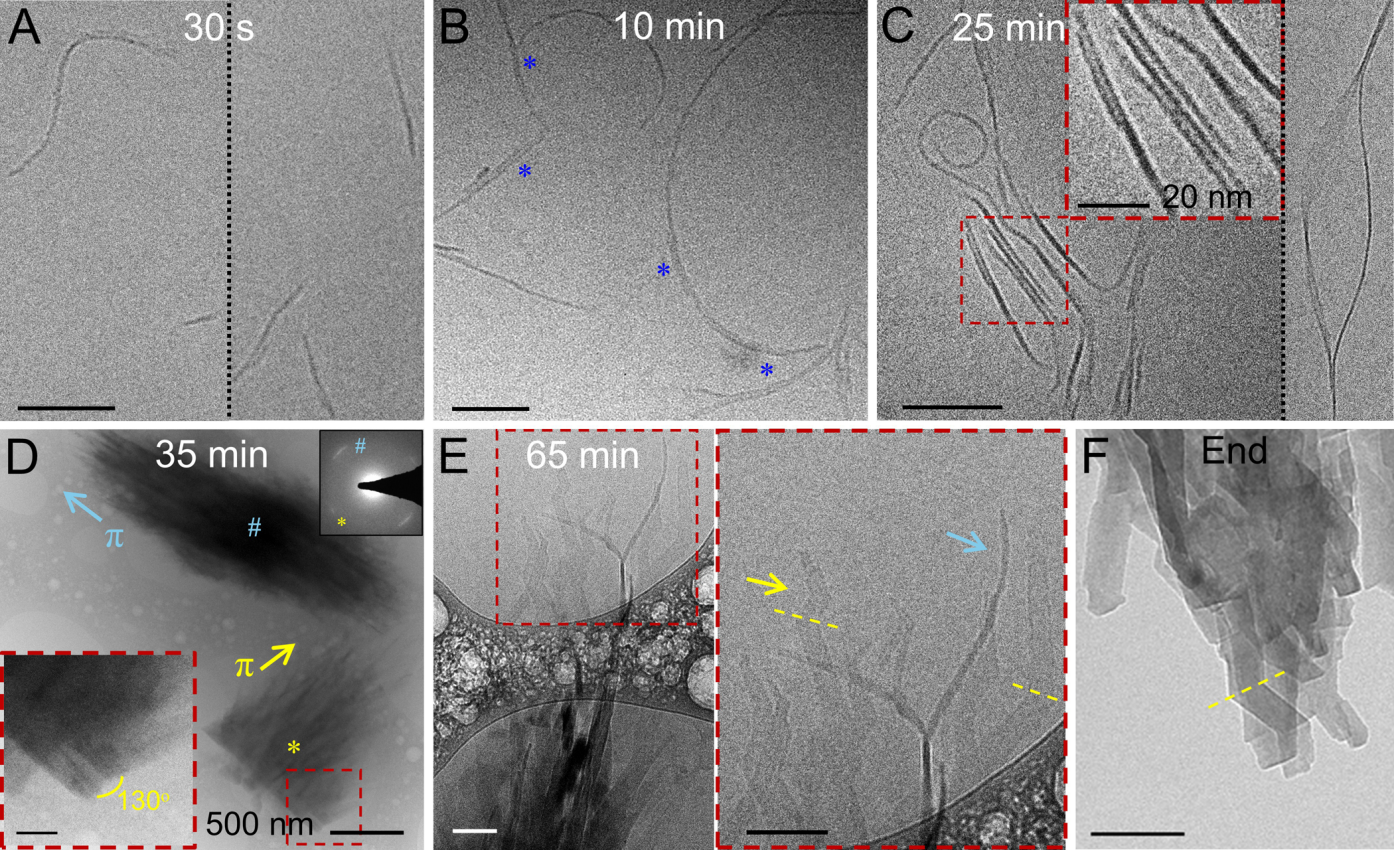
Cryo-TEM micrographs of vitrified guanine solutions during crystallization. (A) 30 s – flexible 1D fibers of varied lengths but conserved width. (B) 10 min – elongation of fibers, blue asterisks: regions exhibiting fusion of two fibers. (C) 25 min – co-oriented assemblies of straightened fibers, inset: magnified view of the fibers aligning parallel to each other. Some fibers exhibit a helical, twisted ribbon morphology (right). (D) 35 min – coalescence of fibers into bundles through a “nearly oriented-attachment” process, left inset: magnified view of the faceted region of the yellow starred bundle, right inset: electron diffraction patterns from the blue and yellow starred bundles exhibiting strong (100) reflections. (E) 65 min – appearance of the “chevron” faceted crystal morphology at the bundle core with fibrous characteristics retained in the periphery, inset: high magnification region of interest, yellow arrow: faceted regions, blue arrow: fibrous regions. (F) TEM micrograph of mature prismatic guanine crystals precipitated at the end of crystallization (*t* = 120 min). Scale bars (unless specified otherwise): 100 nm.

To confirm that these assemblies (Figure 2A–E) are composed of neutral guanine, we precipitated guanine by rapidly quenching solutions from pH 13 to pH 9 (*S* ∼ 8.0), where neutral guanine molecules predominate.^30,42^ The resulting bundles of π-stacked guanine fibers (Figure 3A,B) closely resemble those obtained from gradual self-assembly (Figure 2), indicating that both methods result in the formation of neutral guanine assemblies. Some bundles exhibit a more faceted morphology, with the transformation from a fibrous to faceted character progressing from the core to the periphery (Figure 3C). At more advanced stages (Figure 3D), bundles are composed of thin, faceted crystals devoid of fibrous features, with the emergence of long-range periodicity in the H-bonding (012) plane. Larger prismatic crystals (Figure 3E) emanate from the bundles, indicating that they act as a dense phase from which a 3D-ordered crystal emerges.

**Figure 3:**
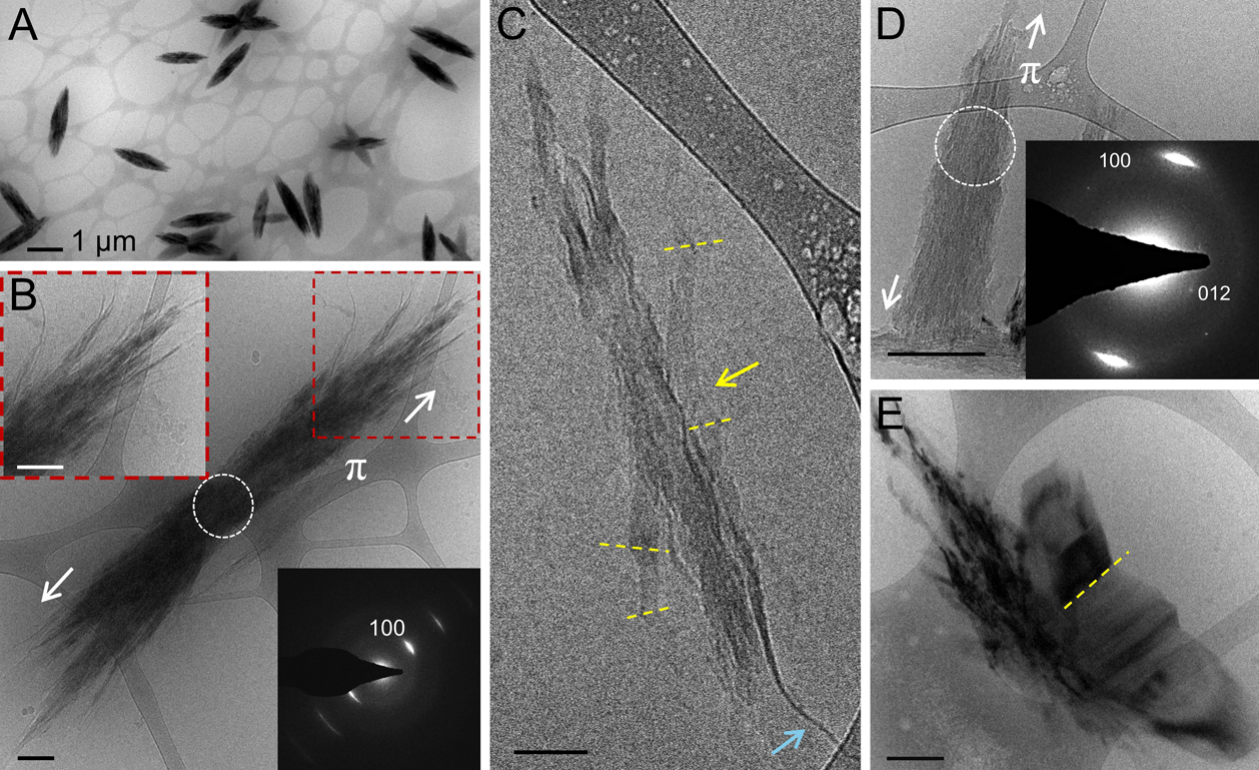
Cryo-TEM micrographs of guanine solutions after rapid quenching. (A) Low magnification overview of the grid showing uniform bundles of π-stacked guanine fibers. (B) A bundle of guanine fibers. Left inset: high magnification of the bundle periphery, highlighting the fibrous nature. Right inset: selected area electron diffraction (SAED) from the bundle core exhibiting intense (100) reflections. (C) A partially transformed bundle exhibiting twinned faceted (yellow dashed lines and yellow arrow) and fibrous (blue arrow) regions. (D) A more mature bundle devoid of any fibrous features depicting the transformation to a more ordered state. Inset: SAED from the center of the bundle exhibiting weak diffraction in the (012) H-bonded plane. (E) A prismatic crystal emerges from a fibrous bundle. Scale bars (B–E): 200 nm.

### Structural Evolution during Guanine Crystallization

*In situ* synchrotron powder X-ray diffraction (PXRD) and Raman spectroscopy revealed structural transformations occurring in bulk crystallizing solutions. Initially, PXRD patterns displayed an amorphous background corresponding to scattering from water (Figure 4A). As crystallization progresses, a single broad peak at d^–1^ ∼ 3.125 nm^–1^ emerges, corresponding to the (100) plane of β-guanine – indicating the onset of long-range ordering along the π-stacking direction. The intensity and sharpness of the (100) peak increase as crystallization progresses and diffraction along the (012), (002), and (011) planes progressively emerges.

**Figure 4:**
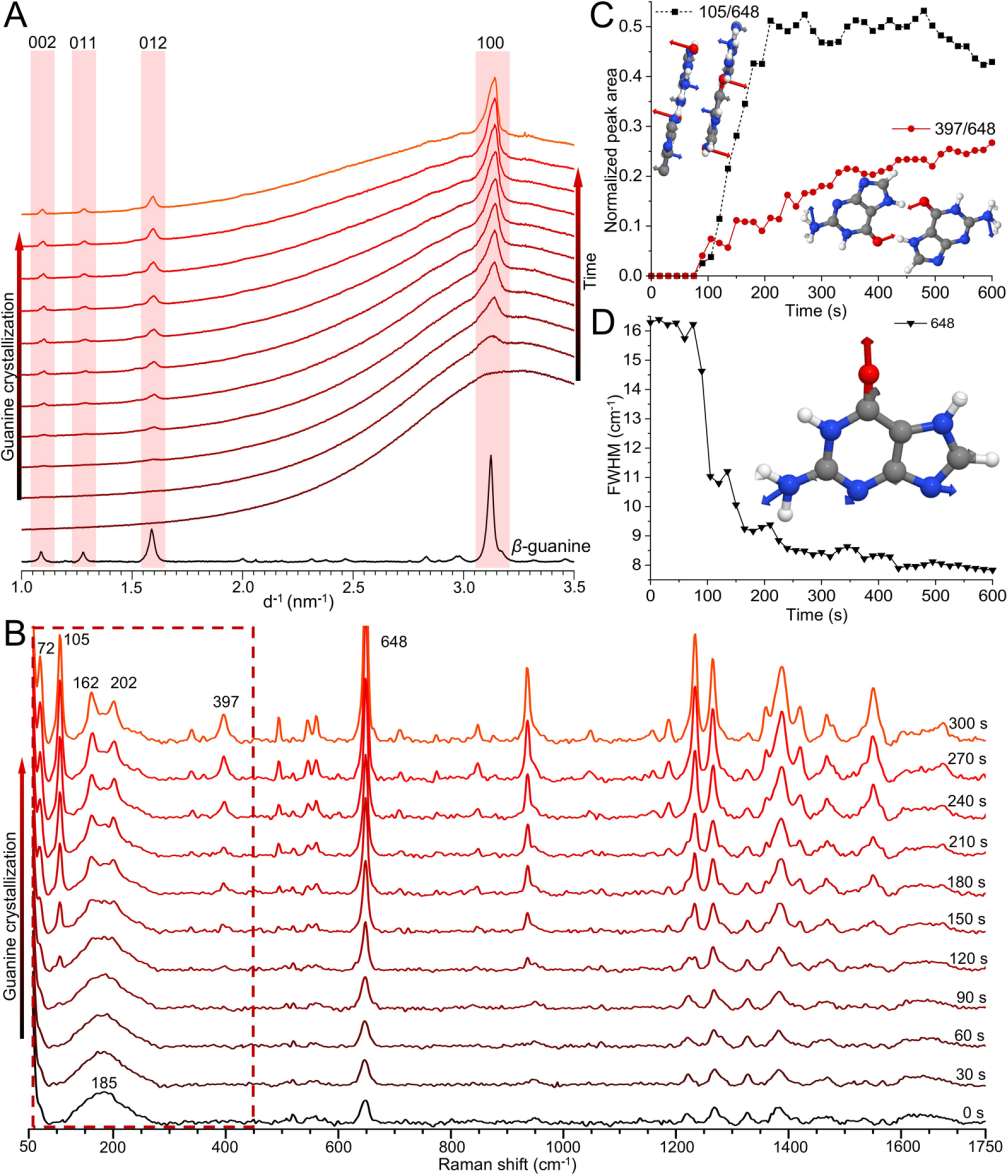
Structural evolution of guanine during crystallization from solution, demonstrating a multi-step crystallization process. (A) PXRD patterns from a crystallizing guanine solution (59.4 mM), collected every 3.5 min. Black trace: β-guanine standard (λ = 0.825 Å). (B) Raman spectra collected from a crystallizing guanine solution (59.4 mM). Dashed red box: the low-frequency region. (C) Peak areas of a Raman π-stacking mode at 105 cm^–1^ (black trace) and a H-bonding mode at 397 cm^–1^ (red trace) as a function of time, normalized against the peak area of the guanine ring-breathing mode at 648 cm^–1^. (D) FWHM of the ring breathing mode as a function of time.

At the early stages of crystallization, “fingerprint” Raman vibrations of molecular guanine are observed, with the intense band at 648 cm^–1^ assigned to the purine ring-breathing mode of guanine^45^ (Figure 4B). In contrast, low-frequency lattice modes are initially absent. The broad peak at 185 cm^–1^ is assigned to the H-bonding of water,^46^ indicating a guanine solution. As crystallization proceeds, lattice mode peaks at 72, 105, 162, 202, and 397 cm^–1^ become visible as the periodic ordering of a guanine solid increases during crystallization. To rationalize these changes, Raman modes of β-guanine were calculated using DFT (Figure S6). Peaks below 300 cm^–1^ are associated with out-of-plane lattice vibrations along the π-stacking axis (Figure S7). The peak at 397 cm^–1^ is assigned to synchronized amino/carbonyl deformations, which are sensitive to perturbations in H-bonding induced by in-plane lattice vibrations.^47^ Plots of the normalized peak areas of the 105 cm^–1^ and 397 cm^–1^ bands versus time (Figure 4C) show that the onset of crystallization (indicated by a sudden decrease in the FWHM of the 648 cm^–1^ band, Figure 4D) is associated with a rapid increase in ordering along the π-stacking (Figure 4C, 105 cm^–1^, “π-stacking peak”). In contrast, there is a slow, progressive increase in ordering along the H-bonding (Figure 4C, 397 cm^–1^, “H-bonding peak”). *In situ* electron microscopy, powder diffraction, and Raman spectroscopy thus reveal a multi-step ordering process during crystallization, where long-range periodicity emerges first along the π-stacking and then in the H-bonded plane (Figure 2D). This mechanism is consistent with the biological crystallization of guanine in spiders, where π-stacked granules transform into 3D periodic crystals via the relaxation of stacking faults and twinning defects.^48^

### MD Simulations of Guanine Self-Assembly during Nucleation

To investigate earlier stages of crystallization, unbiased MD simulations (Movie S1) of guanine (*S* = 5) in an aqueous solution were performed initially for 500 ns (*T* = 300 K, totaling 800,000 atoms). Unlike the crystallization experiments (Figures 2–4), MD simulations were performed at neutral pH, as pH cannot be incorporated into nonreactive force fields required for large-scale simulations. However, since nucleation is driven by the supersaturation of insoluble, uncharged guanine molecules in solution (achieved by modulating pH in the experiments), such simulations likely account for the major dynamic and structural changes occurring during nucleation. In the first few nanoseconds of the simulation, short-lived, single-column clusters of ≤6 stacked guanine molecules form (Figure 5A, 0.5 ns). The preference of nucleotides^49^ and free purines^50^ to stack is crucial to the stabilization of nucleic acid conformations.^51^ Stacking minimizes the energy of the system by reducing the exposure of hydrophobic molecular faces to water while leaving the hydrophilic moieties on the edges free to engage in hydrogen bonding with the solvent. These stacks then merge into long-lived two-column “nuclei” (Figure 5B, 2.5 ns), comprised of alternating triple and double H-bonded pairs, an arrangement which maximizes the number of H-bonds *within* a layer and the π-overlap *between* layers (Figure 5B’, S8). The double H-bond motif in these two-column nuclei is different from that in the β-guanine crystal, forming over the dihedral angle between O6, N7 and N1, O6, rather than the wider angle between O6, N7 and O6, N7 (Figure 5B’). Within 20 ns, these nuclei grow along the π-stacking direction by single-molecule addition (Figure S9) and occasionally, in the H-bonded plane, by lateral attachment with other columnar stacks. The alternative H-bonding arrangement in the two-column nuclei means that when two such nuclei merge (Figure 5C), the arrangement of the four in-plane guanine molecules differs from the β-guanine structure in containing a “defective” guanine, which is inverted with respect to the crystal structure (Figure 5C’). By 20 ns, Ostwald ripening^52^ (Figure S10) results in the formation of three large fibers, typically four molecules wide (diameter ∼15Å, Figure S11) containing ∼100 molecules (Figures 5C and S10). Within these fibers, the “defective” guanine molecule bends out-of-plane due to steric repulsion between amine groups, yielding a helix (Figure S12). When two helices merge, the structure converges to planar β-guanine (Figure S13), as the defective guanine is incorporated into the H-bonded network of an adjacent layer. This “healing” process indicates that different H-bonding arrangements are favored in different size regimes – a key feature of multi-step nucleation.

**Figure 5:**
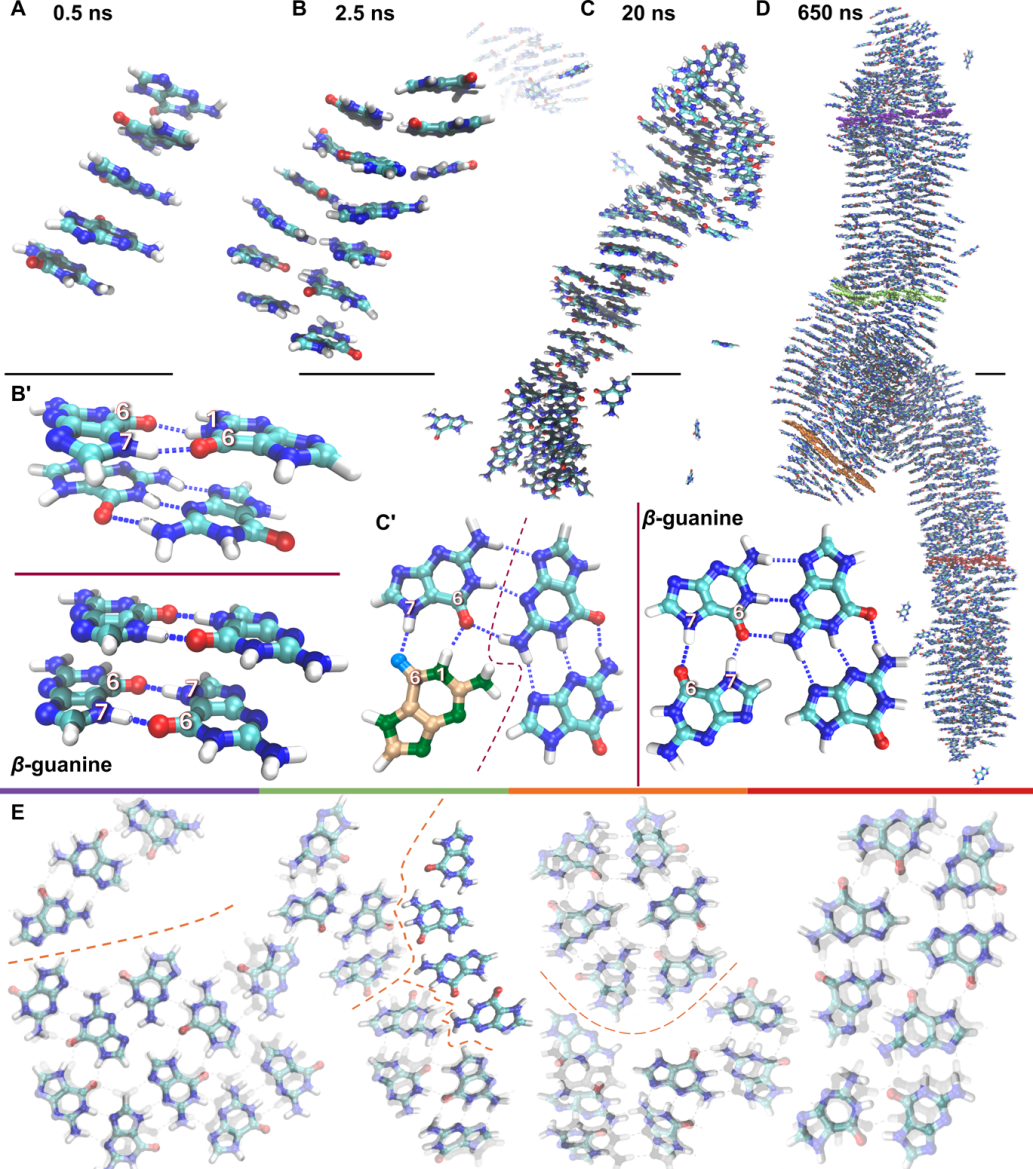
MD simulations of guanine nucleation. Representative snapshots of the largest guanine clusters at (A) 0.5 ns – single-column clusters of guanine molecules, (B) 2.5 ns – two-column “nuclei”, (C) 20 ns – guanine fiber growth via “particle attachment”, and (D) 650 ns – 25 nm long guanine fiber. (B’) Comparison of the H-bonding arrangement in the two-column nuclei at 2.5 ns (top) and β-guanine (bottom). (C’) Comparison of the in-plane arrangement of the four-molecule wide guanine fiber at 20 ns (left, “defective” guanine: gold-green colored), and β-guanine (right). (E) Examples of the in-plane molecular arrangement in cross sections through the final fiber at 650 ns, overlaid over the β-guanine structure (transparent). Colored bars match the overlays to the highlighted layers in the fiber (D). Orange dashed lines: domain boundaries. Scale bars (A–D): 1 nm.

After 20 ns, the fibers elongate and widen by further attachment events (Figure S9). This occurs mostly in the π-stacking direction until at 500 ns, one large fiber remains (Figure S10). This fiber was transferred to a larger box (1.5 M atoms) and further equilibrated to assess its stability in the absence of finite-size effects induced by 3D periodic boundary conditions. The resulting 25 nm-long fiber (Figure 5D) has a size and aspect ratio of ∼7 (Figure S14), comparable to the smallest fibers observed experimentally (Figure 2A). It is composed of misoriented domains exhibiting an exact H-bonded arrangement of β-guanine (*P*2_1_/*c*), separated by domain boundaries (Figure 5E). The close match between the simulated structure of the fiber and the guanine crystal structure also serves as a validation of the chosen force field, increasing our confidence in the mechanistic insights gained at the molecular scale.

### Quantitative Analysis of Structural Ordering during Nucleation

To quantify the ordering of guanine during nucleation, we measured the relative in-plane orientation between neighboring guanine molecules (φ and θ, Figures S15 and S16) and their stacking distances, *d*_π_ – represented by a probability heatmap of preferred pairwise orientations (Figure 6). The angular in-plane distribution for an ordered β-guanine crystal is characterized by four maxima (Figure 6A, annotated with the corresponding structural motifs). The fiber obtained after 650 ns (Figure 5D) possesses an in-plane distribution similar to that of the β-guanine crystal (Figure 6B). However, the repeating unit peak (at 0°, 0°) is more diffuse due to domain boundaries in the fiber (Figure 5D, E). The nonbonded motif (at 130°, 50°) is the most diffuse, as it corresponds to the weakest pair interaction and is further diluted by the defective guanine (Figure 5C’) peak expected at ∼170°,25°. For an ordered β-guanine crystal, these four in-plane maxima repeat every ∼3.1 Å along the stacking direction (Figure 6C). However, the fiber exhibits a random molecular arrangement between layers (indicated by the crisscrossed pattern; Figures 6D and S16). We quantified the evolution of these distributions over time by evaluating the Kullback–Leibler divergence^53,54^ – a measure of statistical difference between the molecular distributions in the fiber and those in the β-guanine crystal (Figure 6E). Within the first 100 ns, short-range, in-plane ordering of guanine rapidly emerges and then slowly converges to that of the β-guanine crystal over a few hundred nanoseconds, with small spikes occurring during “merging events” that create new domain boundaries (in close agreement with the qualitative description in Figures 5 and S13). The emergence of orientational ordering *between* H-bonded layers does not occur on the time scale of this simulation. This is because the H-bonded layers of β-guanine have a constant offset along the stacking direction, which cannot be achieved in the finite-width fiber. This is also consistent with *in situ* spectroscopy and diffraction (Figures 2–4), showing that long-range periodicity in the H-bonding plane only emerges after numerous fibers attach into bundles, forming a sufficiently large and dense phase for 3D-ordered crystals to emerge over the course of minutes (Figure 4). It should be noted that the α- and β-polymorphs differ only in their stacking and possess the same in-plane order. Therefore, they cannot be distinguished on the nanometer scale accessible to simulations.

**Figure 6:**
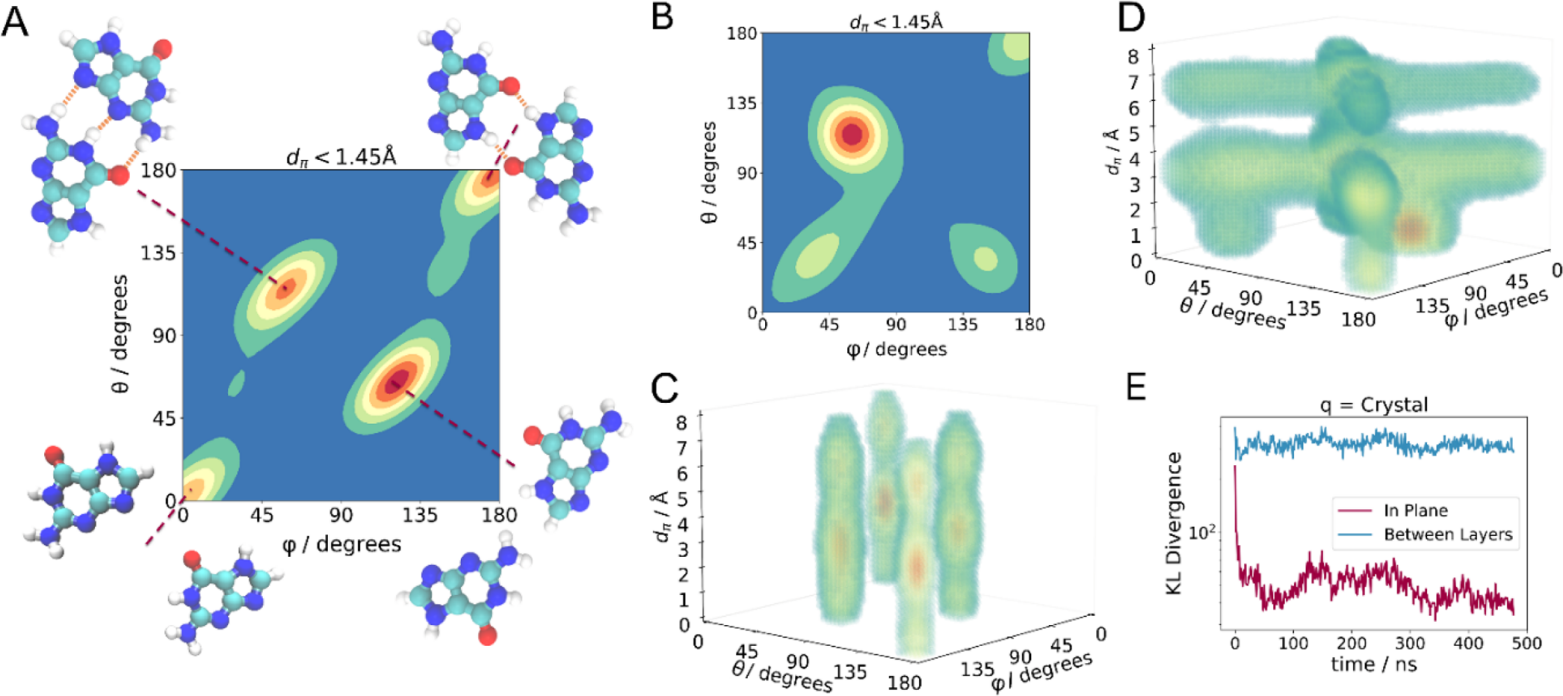
Quantification of guanine ordering during nucleation. Probability heatmap of the preferred pairwise in-plane distribution of guanine molecules as a function of angles φ and θ in (A) the β-guanine crystal, and (B) the simulated fiber at 650 ns (Figure 5D). Structural motifs corresponding to the peaks are labeled: “triple H-bond” (50°, 130°), “double H-bond” (180°, 180°), “repeating unit” (0°, 0°), and ″non-bonded motif″ (130°, 50°). The distribution of angles φ and θ resolved along the stacking distance, *d*_π_ in (C) the β-guanine crystal, and (D) the simulated fiber at 650 ns. (E) Temporal evolution of Kullback–Leibler divergence,^54^ quantifying the similarity in distributions of in-plane and stacking arrangements between the β-guanine crystal and the simulated fiber (Figure 5D).

## Conclusions and Outlook

Despite their widespread use in pharmaceuticals, materials, and biology, the crystallization pathways of small organic molecules are poorly understood. The time scales and rarity of nucleation events mean that without sampling techniques, MD simulations of nucleation from solution are usually impossible or lack experimental verification.^18,55^ On the other hand, direct observations of crystallization by microscopy are typically confined to morphological-level descriptions. Here, unbiased MD simulations and *in situ* experiments converge to reveal a multi-step crystallization mechanism of guanine from aqueous solution. We suspect that similar mechanisms underlie the crystallization of many other planar aromatic molecules that assemble through H-bonding and π-stacking. This paves the way for applying similar techniques to probe the crystallization mechanisms of other purine^56^ and pteridine^21^ biogenic crystals and artificial molecular materials.^57,58^ MD simulations may also be used to understand how small molecule dopants present in biological crystals influence crystal nucleation and growth.

## Supplementary Material







## Notes

Supplementary cryo-TEM images, XRD patterns, Raman spectra, DFT calculated Raman vibration modes, MD simulation snapshots, and quantitative analysis data (PDF)
Timelapse of the 500 ns long simulation of a supersaturated guanine solution in water (Figure 4A–C), where the blue lines indicate the simulation box, and the grey renderings represent the periodic images of the box in two out of the three dimensions (Movie S1) (MP4)
Timelapse of the 150 ns long simulation of the fiber grown in Movie SM1 in a two times larger box (Figure 4D), where the blue lines indicate the simulation box; the resulting fiber obtained at the end of 500 ns of simulation (Movie S1) was transferred to a larger box (1.5 million atoms) and it was equilibrated for further 150 ns to assess its stability in the absence of finite-size effects induced by 3D periodic boundary conditions (Movie S2) (MP4)
