# Supplementary material for: Guanine Crystallization by Particle Attachment
Source: J Am Chem Soc. 2025 May 23;147(22):19139–47. doi: 10.1021/jacs.5c04543 (PMC12147113; doi:10.1021/jacs.5c04543)
Supplement: Supplementary file 1 [file ja5c04543_si_001.pdf]

Methods and Supplementary Information:

Guanine Crystallization by Particle Attachment

Shashanka S. Indri,[1]† Florian M. Dietrich,[2]† Avital Wagner,[1] Michal Hartstein,[3] Einat Nativ-Roth,[4] Mariela J. Pavan,[4] Leeor Kronik,[3] Matteo Salvalaglio,[2]* Benjamin A. Palmer.[1]*

[1]Department of Chemistry, Ben-Gurion University of the Negev, Be'er Sheba, 8410501, Israel.
[2]Department of Chemical Engineering, University College London, London WC1E 7JE, United Kingdom.
[3]Department of Molecular Chemistry and Materials Science, Weizmann Institute of Science, Rehovoth 7610001, Israel.
[4]Ilse Katz Institute for Nanoscale Science and Technology, Ben-Gurion University of the Negev, Be'er Sheba 8410501, Israel.
†These authors contributed equally.
*Corresponding authors: m.salvalaglio@ucl.ac.uk, bpalmer@bgu.ac.il

M1. Methods

Guanine crystallization

For the crystallization of mature guanine crystals from aqueous solutions (Fig. 2F), a method similar to the one reported in Gur et. al.,[1] was adopted. 20 mg of guanine powder (Sigma-Aldrich) was dissolved in a 20 mL solution (6.6 mM) of 0.1 M NaOH (pH 13). Crystallization was induced by titrating the solution with 0.1 M HCl using an auto-titrator (TitroLine 7750, SI Analytics) until the pH reached 11.35. The titration rate was set to 0.1 mL per minute. The obtained crystals were collected after two hours and cleaned by three rounds of centrifugation with DDW and then imaged with TEM (Fig. 2F). The experiments were replicated three times with similar results. The aqueous solubility of guanine at pH 7 and 25°C is 25.4 μM.[2]

For the gradual self-assembly crystallization experiments (Figs. 2 A-E, S4), 20 mg guanine was dissolved in 20 mL solution (6.6 mM) of 0.1 M NaOH (pH 13). Crystallization was induced by titrating the solution with 0.1 M HCl using an auto-titrator until pH 11.35. Then at various time points, aliquots of the crystallization solution were vitrified until the solution became cloudy and guanine had precipitated. For rapidly quenched samples (Fig. 3), 1 mL of 6.6 mM guanine solution (pH 13) was quenched with 0.1M (0.92 mL) HCl using a pipette and aliquots of the crystallization solution were vitrified instantly.

Cryo-transmission electron microscopy (cryo-TEM)

Vitrified samples of crystallization solutions (Figs. 2,3, S1, S4) were prepared on a copper grid coated with a perforated lacey carbon 300 mesh (Ted Pella Inc.). A 2.5μL droplet from the solution was applied to the grid and blotted with a filter paper to form a thin liquid film of solution. The blotted samples were immediately plunged into liquid ethane at its freezing point (-183°C). The procedure was performed automatically in the Plunger (Leica EM GP2). The vitrified specimens were then transferred into liquid nitrogen for storage. The samples were studied using a Talos F200C TEM Thermo Fisher Scientific, at 200kV maintained at -180 °C; and images and electron diffraction patterns were recorded on a Ceta 16M camera (4k × 4k CMOS sensor) from Thermo Fisher Scientific at low dose conditions, to minimize electron beam radiation damage.

Transmission electron microscopy (TEM)

For *ex situ* TEM measurements (Fig. 2F), guanine crystals obtained at the end of crystallization were cleaned using DDW and resuspended in DDW. 3 μL of the resulting suspension was dropped on a glow-discharged Cu meshed TEM grid and allowed to dry. The resulting samples were imaged with a Thermo Fisher Scientific (FEI) Tecnai T12 G^2 TWIN TEM operating at 120 kV. Images and electron diffraction patterns were recorded using a Gatan 794 MultiScan CCD camera. The images were recorded with consideration of potential beam damage to the sample, thus appropriate illumination conditions (e.g., spot size) were used to avoid beam damage

In situ X-ray diffraction

In situ X-Ray diffraction measurements (Fig. 4A) were recorded on the Materials Science/X-ray Powder Diffraction beamline at the SESAME (Jordan) using a radiation wavelength of 0.825 Å. For each measurement, the sample was placed at a distance of 740.4 mm from a Pilatus 300 K area detector with 172 μm pixel size. The area detector covers 6.4°/frame at a 740.4 mm distance from the sample and was used to collect diffraction patterns from 3.3° to 21° (in 3 frames). Average exposure time was 60 seconds/frame, with another 7.5 seconds between each frame, totaling to 3 minutes and 15 seconds per pattern. There was another 15 seconds of resting time between each measurement.

The measurement was performed on a droplet of guanine solution of 9mg/mL (59.4 mM at pH 13) placed on a glass slide. Data were collected in reflection mode at room temperature. A NIST (640f) Si standard was used to calibrate the instrument, while the lattice parameter of Si was used to determine the exact wavelength during the measurements. The detector was set to collect a diffraction image every 6°, while the collected images were then processed to extract the merged diffraction pattern through an image J scripting mode.

Powder Xray diffraction

Powder Xray diffraction measurements (Fig. S5) were carried out on a Panalytical Empyrean powder diffractometer equipped with a position-sensitive detector X'Celerator. The scanning spanned 2θ values from in the 5–40° range (approximately 10 minutes acquisition time) using Cu Kα radiation (λ = 1.54178 Å) at 40 kV and 30 mA.

***In situ* Raman spectroscopy**

Micro-Raman measurements (Figs. 4B-D, S6) were performed with a confocal Horiba LabRam HR Evolution, equipped with a Syncerity-OE CCD detector (deep-cooled to – 60 ºC, 1024 x 256 pixels). The excitation source was a 532 nm laser with a power on the sample of 35 mW. The laser was focused with a 50x LWD objective (Olympus LMPlanFL-N, NA = 0.5, spot size ~ 1.3 µm). The measurements were taken using a 600 g mm^{-1} grating and a 100 µm confocal hole. The Raman was measured in a droplet of 9 mg/ml guanine solution (59.4 mM at pH 13) placed on an Au sputtered surface at 10 ºC using a Linkam THMS600 cell. A time map was programmed to collect Raman spectra every 3s over a period of 600 s, with an exposure time of 3s seconds per spectrum. The spectra were baseline correction after dividing the raw data by the Bose-Einstein factor[3]. The spectra were measured and processed using LabSpec 6, version 6.5.1.24 and OriginPro.

DFT calculations of Raman vibrational modes

All calculations (Figs. S6, S7) were performed within the framework of DFT, using the Vienna *ab initio* simulation package (VASP) version 6.3.1[4] and the Phonopy simulation package[5,6]. All calculations were executed with the Perdew-Burke-Ernzerhof (PBE) exchange and correlation functional[7] and the DFT-D3 method was performed with the Becke-Johnson damping function[8,9]. In several cases, this approximation has been shown to produce useful outcomes for predicting low frequency Raman modes of organic molecular crystals at a reasonable computational cost[10,11]. A plane-wave cutoff of 1000 eV was used in all calculations.

Full atomic relaxations were performed to the previously calculated structure of *β*-guanine[12], with a threshold of 10−3 eV $Å^{-1}$ for the residual forces and a k-point grid sampling of the Brillouin zone of 6×2×1. The finite differences method was used to calculate phonon frequencies, with both VASP and Phonopy. The atomic displacement width used was 0.01 Å. The resulting phonon eigenfrequencies and eigenvectors were used to simulate the Raman spectrum. The isotropic Raman intensities were calculated using the Porezag and Pederson method[13], which was implemented in a Python code[14]. The code uses VASP to calculate the macroscopic dielectric tensor with respect to each phonon mode. To obtain a broadened spectrum similar to the experiment, we applied a Lorentzian broadening on the calculated spectrum, with a width of 2 cm^{-1}.

MD simulations

All simulations (Figs. 5, S8-S14) were performed using GROMACS version 2023.2[15]. The forcefield for guanine was parametrized using AmberTools24[16] and for water the TIP3P force-field was used[17].
For the main simulation (Fig. 5A-C) 914 guanine molecules were solvated with 256,072 water molecules in a cubic box of length 20 nm. The energy of the resulting box was minimized with 5000 steepest descent steps. The box was then equilibrated for 100 picoseconds (ps) in the NVT ensemble to 300 K using GROMAC's V-rescale thermostat and then further equilibrated for 100 ps to 1 bar in a short NPT simulation using a Parrinello-Rahman barostat. This was followed by the actual production run for close to 500 nanoseconds (ns) with a timestep of 2 femtoseconds (fs), a coulomb, vdW and neighborlist cutoff radius of 1.2 nm and PME electrostatics. The simulation was tempered to 300 K using the V-rescale thermostat using a coupling constant of 0.1 ps. The pressure was constrained to 1 bar using a Parrinello-Rahman barostat with a coupling constant of 2 ps.
Afterwards the large semi-crystalline fiber produced from the 500 ns simulation was extracted and transferred to a larger cubic box with a box length of 25 nm and resolvated with additional free guanine (1193 molecules total) and 505,098 water molecules. The simulation was then continued for an additional 150 ns using the same production protocol as above (Fig. 5D).

A reference β-guanine crystal[18] of 512 molecules was equilibrated using the same equilibration protocol as mentioned above and simulated for 100 ns for comparative quantitative analysis (Fig. 6).

Quantitative analysis of MD simulations
For the cluster analysis (Fig. 6, S9, S10) two molecules were considered "connected" if their centers of mass were within 0.9 nm of each other and their π-systems were parallel to each other within ±45°. From those pairwise connectivities, an adjacency matrix was built using the MDAnalysis package[19,20]. The largest cluster was defined as the number of nodes in the largest connected subgraph in the adjacency matrix which was extracted using the package NetworkX[21].
The relative orientation between two guanine molecules is described by assigning each molecule two vectors v_1 and v_2 (Fig. S15). The angle φ is obtained by projecting v_1 of the second molecule into the plane of the first and evaluating the resulting angle,
$$v_{1,proj} = v_{1,2} - (v_{1,2} \cdot n_1) \times n_1$$
$$cos\,\varphi = \frac{v_{1,1} \cdot v_{1,proj}}{|v_{1,1}||v_{1,proj}|},$$
where n_1 is the surface normal of the π-system of molecule one. θ is calculated the same way but using the vectors v_2.
The stacking distance d_π is defined as the distance between the two center of masses projected onto the surface normal of the central molecule,
$$d_\pi = ||(d_{1,2} \cdot n_1) n_1||.$$
The distributions of angles are constructed through kernel density estimation (KDE) using gaussian kernels using the SciPy package[22]. The difference between a distribution p and a reference distribution q was evaluated by implementing a Kullbach-Leibler divergence,[23]
$$D_{KL} = \int_0^{2\pi} q(\varphi\theta)\, log \frac{q(\varphi\theta)}{(p(\varphi\theta) + q(\varphi\theta))/2} \mathrm{d}\varphi\theta.$$

S1. Supplementary experimental results

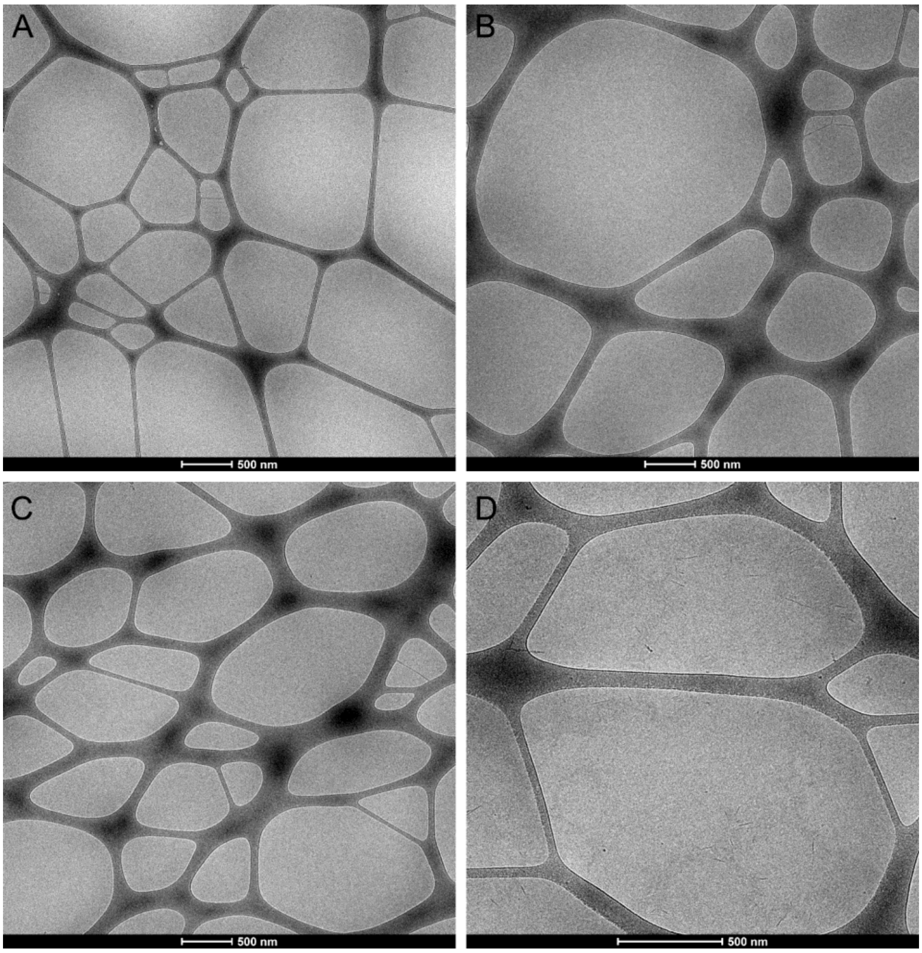

Figure S1. Cryo-TEM micrographs of vitrified samples of **(A)** a 0.1 M NaOH solution, **(B)** a 0.1 M HCl solution, **(C-D)** 6.6 mM guanine solutions at pH 13.00 (0.1 M NaOH). In (C) the grid is completely empty. (D) Sometimes, at later time points (30 minutes and more), guanine fibers were observed.

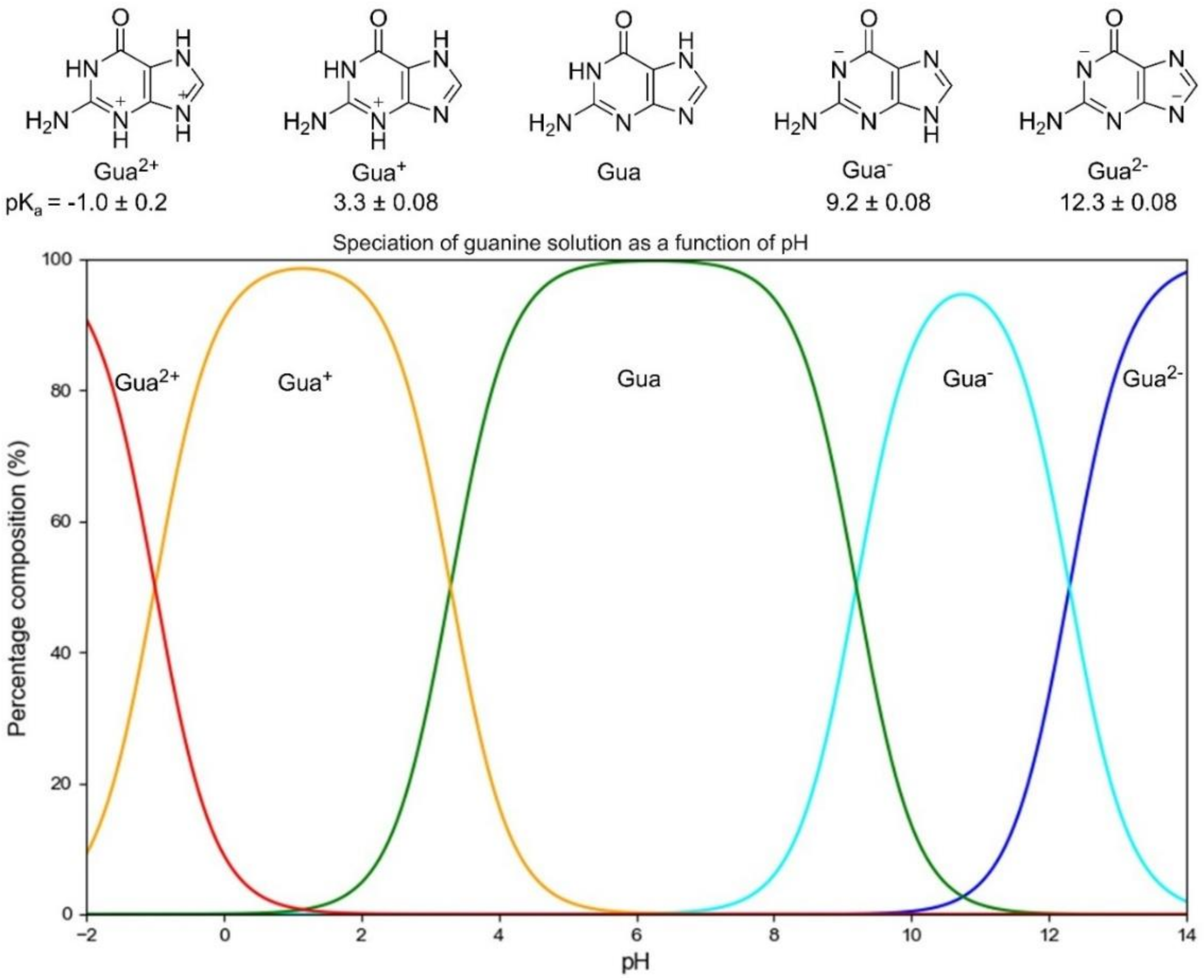

Figure S2. Top: Protonation states of guanine with corresponding pK_a values. Bottom: Speciation of guanine solution (%) as a function of pH, calculated from the pK_a values.

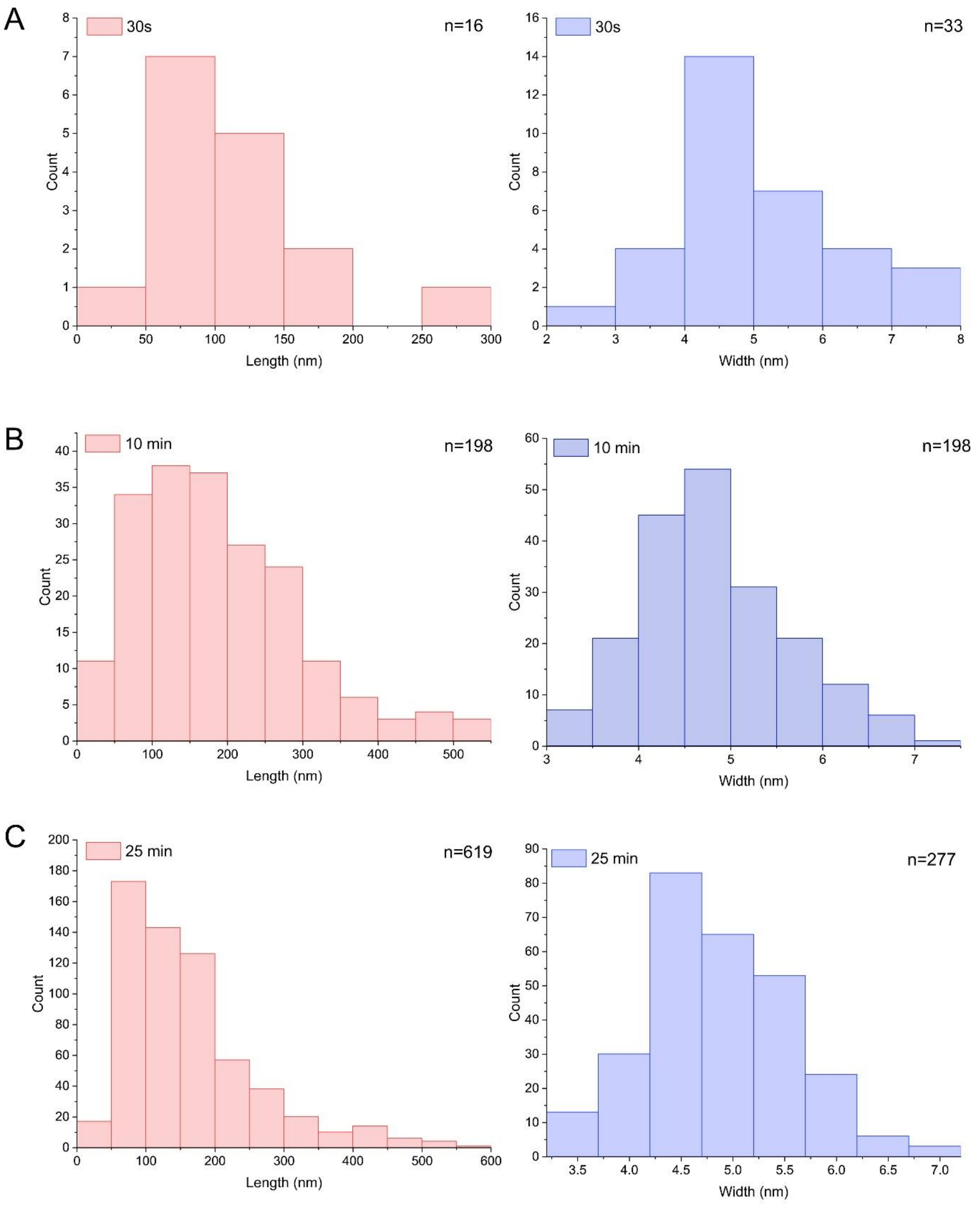

Figure S3. Histograms of the lengths (red, left) and widths (blue, right) of the guanine fibers measured from the cryo-TEM images of the vitrified guanine solution samples at different time points: **(A)** 30 seconds (Fig. 2A), **(B)** 10 minutes (Fig. 2B) and **(C)** 25 minutes (Fig. 2C). n refers to the number of fibers sampled.

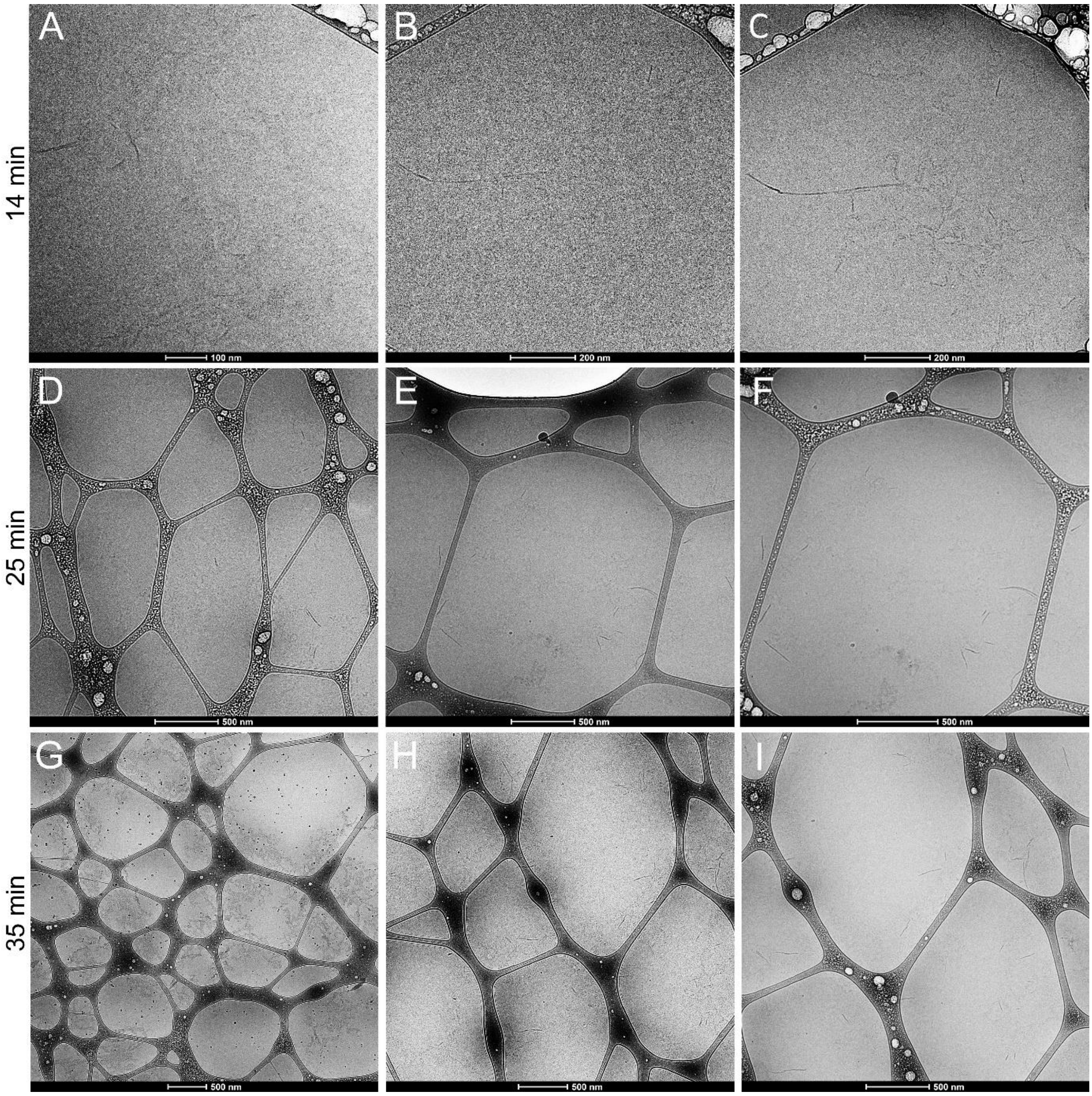

Figure S4. Cryo-TEM micrographs of vitrified guanine solutions showing the beam sensitive, denser phase with granulated features composed of 2-3 nm particles (resembling CaPO4 pre-nucleation clusters (PNCs)[24,25]). Guanine fibers were observed emerging from the denser phase at different time points after lowering the solution to pH 11.35. **(A-C)** 14 mins **(D-F)** 25 mins and **(G-I)** 35 mins. The supposed PNCs are beam sensitive and burn-out/dissolve after taking one image as evidenced in (B→C), (E→F) and (H→I).

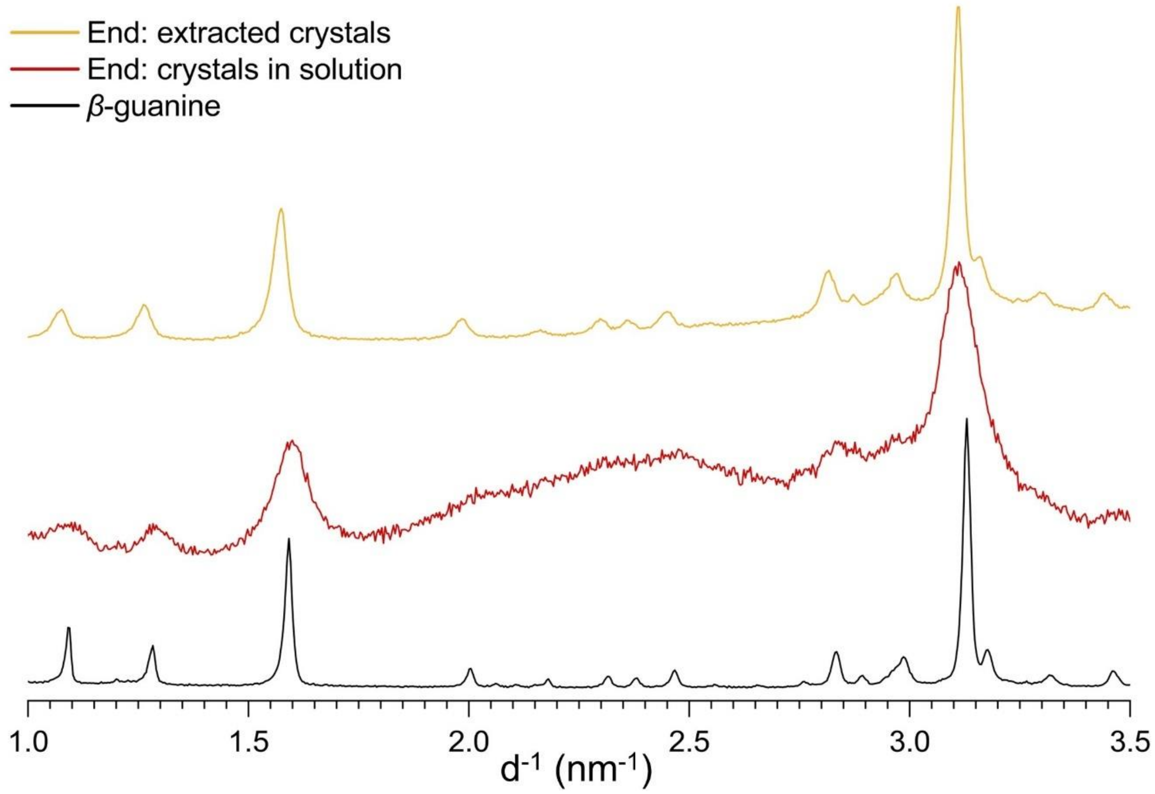

Figure S5. Powder X-ray diffraction patterns of β-guanine (black), the crystallization solution at the end (t = 120 min) of guanine crystallization (red) referenced in Fig. 2 and extracted crystals at the end of the crystallization process (yellow, Fig. 2F).

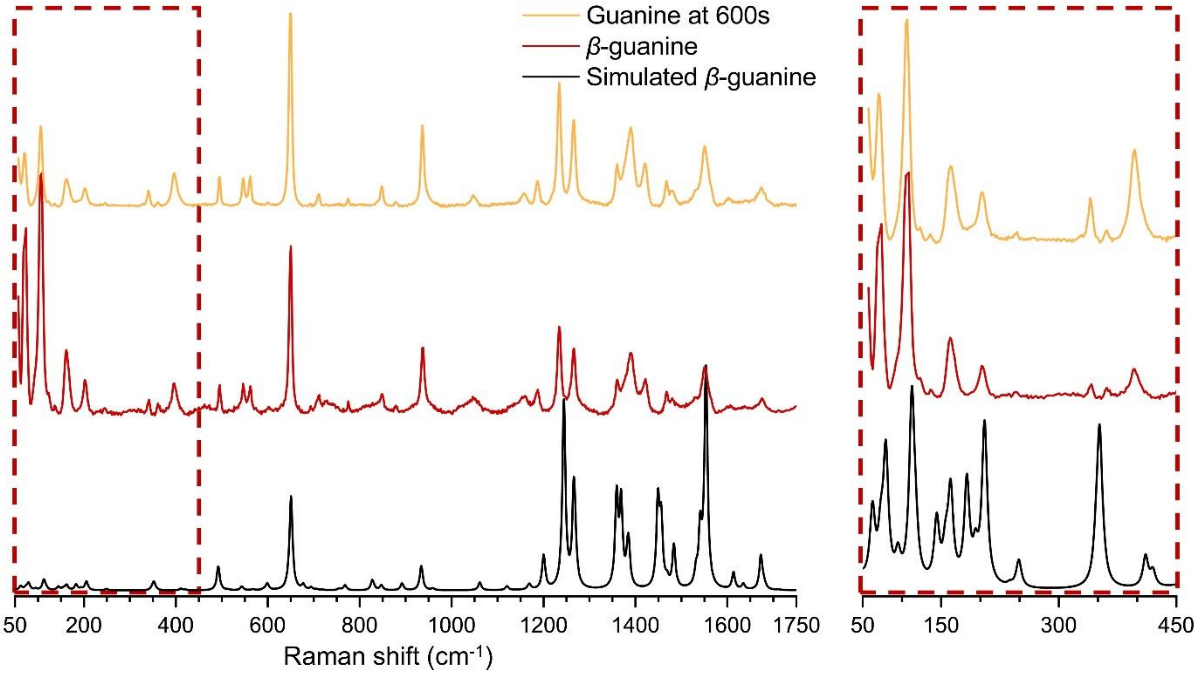

Figure S6. Raman spectra of the guanine crystallized (Fig. 4B) at 600s (yellow), reference β-guanine (red), DFT simulated β-guanine (black). Right (in dashed red box): low frequency region of the spectra.

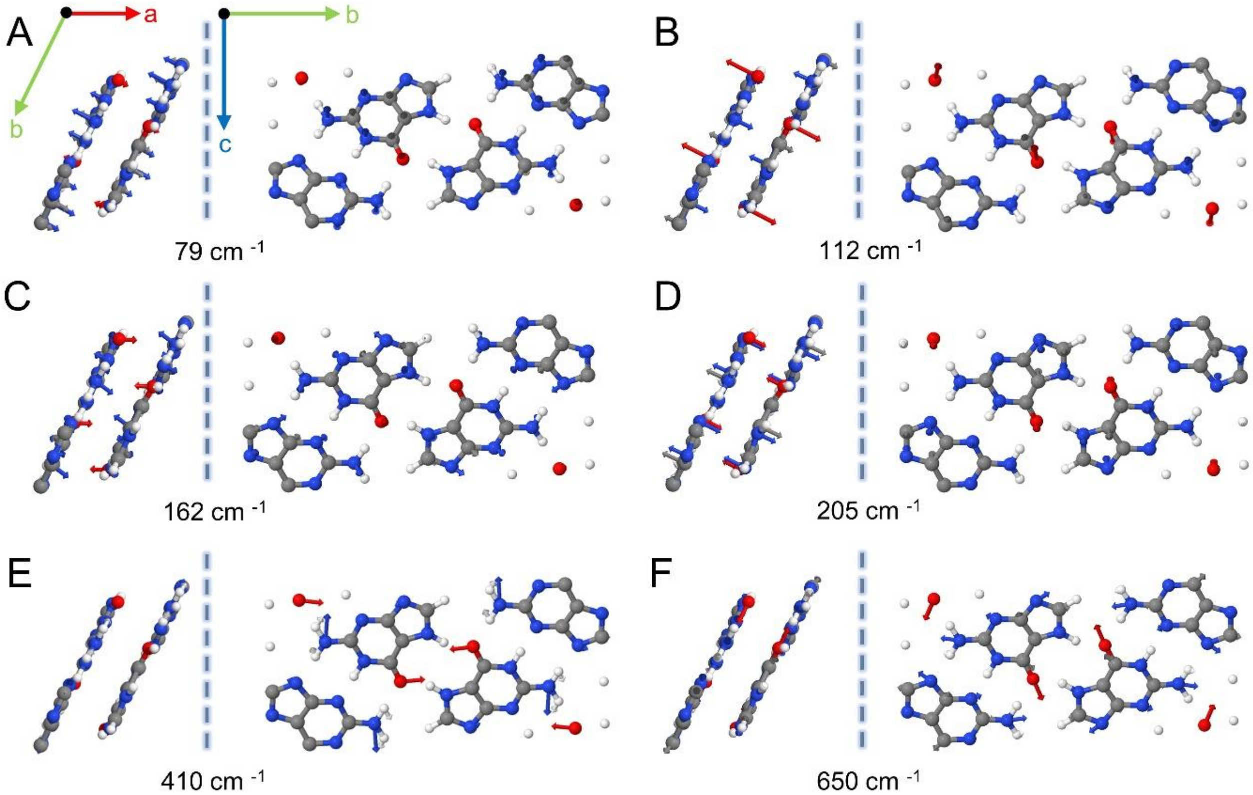

Figure S7. Calculated Raman vibration modes of a β-guanine crystal **(A)** 79, **(B)** 112, **(C)** 162, **(D)** 205, **(E)** 410 and **(F)** 650 cm^{-1} assigned to their corresponding experimental Raman peaks of (A) 72, (B) 105, (C) 162, (D) 202, (E) 397 and (F) 648 cm^{-1} respectively (Figs. 4B-D). Left side represents the *ab* plane and the right side represents *bc* plane of the β-guanine crystal structure. Arrows indicate atomic displacement vectors for a vibrational mode, visualized using Jmol.[3]

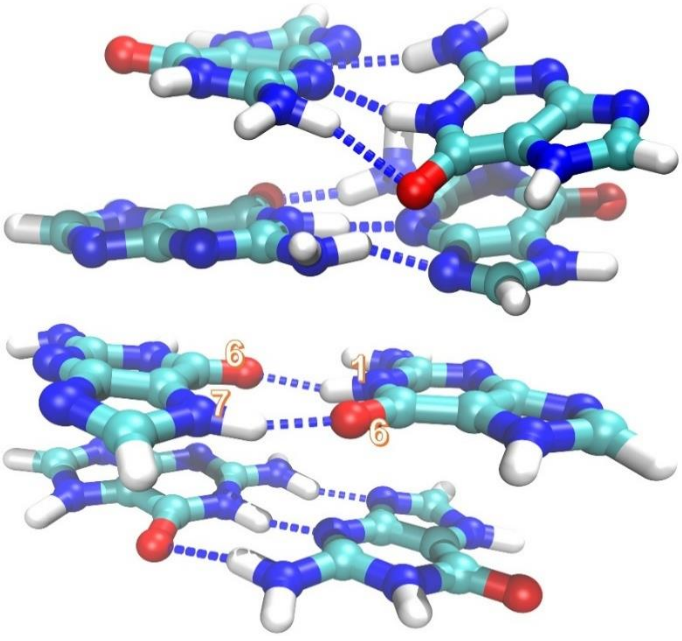

Figure S8. Two-column clusters (Figs. 5B, B') comprised of alternating triple and double H-bonded guanine pairs. The tension between the stacking motifs results from the inherent competition between maximizing intra-layer H-bonding and inter-layer π-overlap.

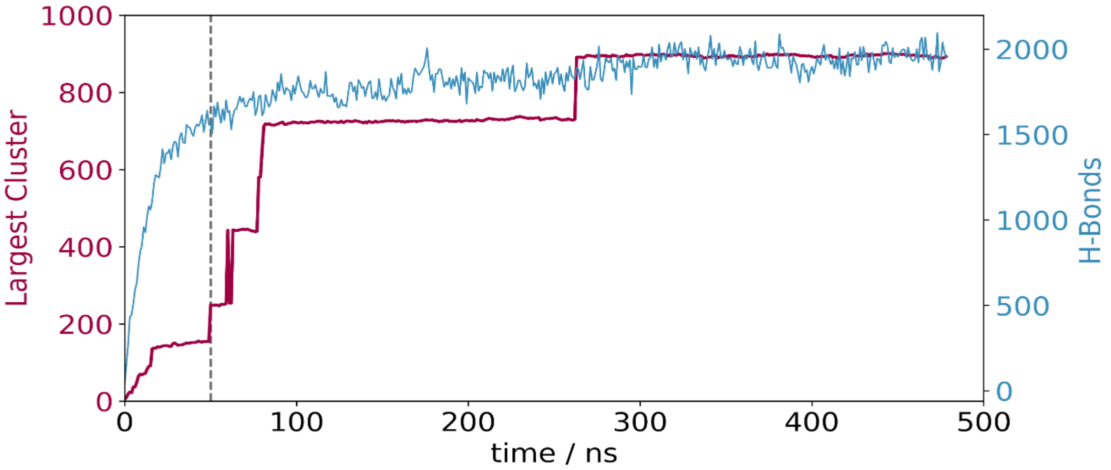

Figure S9. Size evolution of the largest cluster and the number of intermolecular H-bonds between guanine molecules as a function of time over the course of the simulation (Fig. 5). The dashed grey line marks the time at which the solution reaches the equilibrium guanine concentration. Beyond that point, growth occurs only through merging of clusters. Before that point, the cluster also grows gradually via single molecule addition.

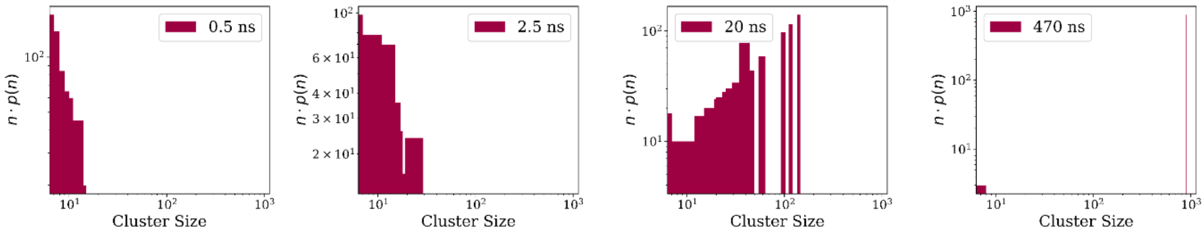

Figure S10. Cluster size distributions over the course of the simulation corresponding to the time steps (Figs. 5A-D).

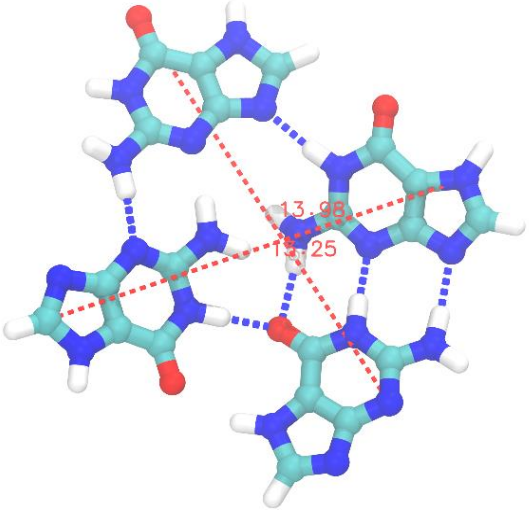

Figure S11. Cross-section of a four-wide guanine column (Fig. 5C) with annotated measurements.

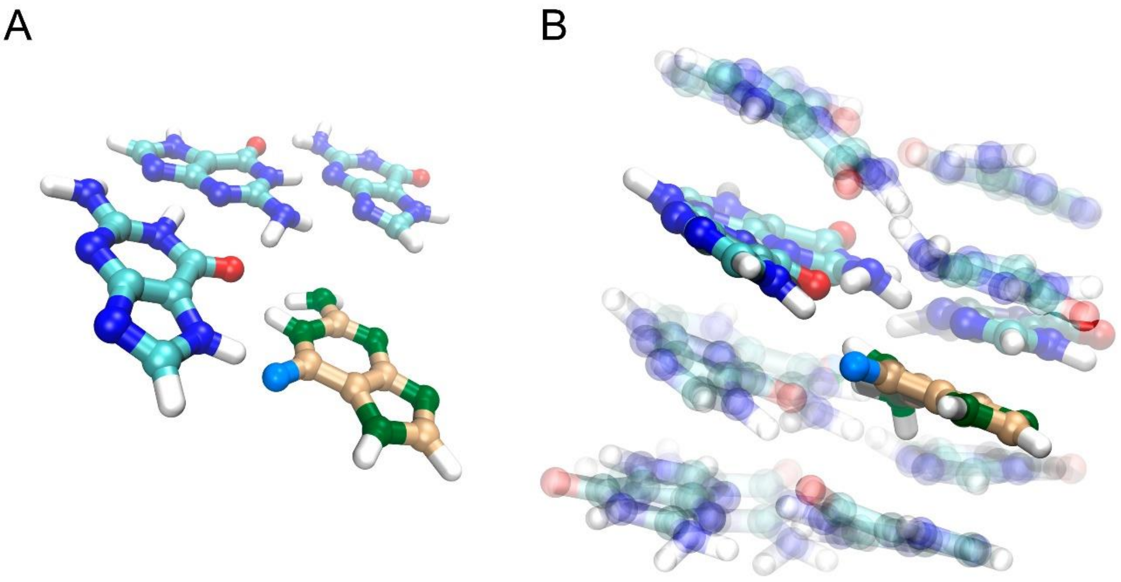

Figure S12. The defective guanine (Figs. 5C, C') bends out of plane in conjunction with the corresponding molecules in adjacent layers, inducing a helical structure. **(A)** In-plane view showing the bending motion. **(B)** Longitudinal-section (LS) view of the motion and the resulting helix.

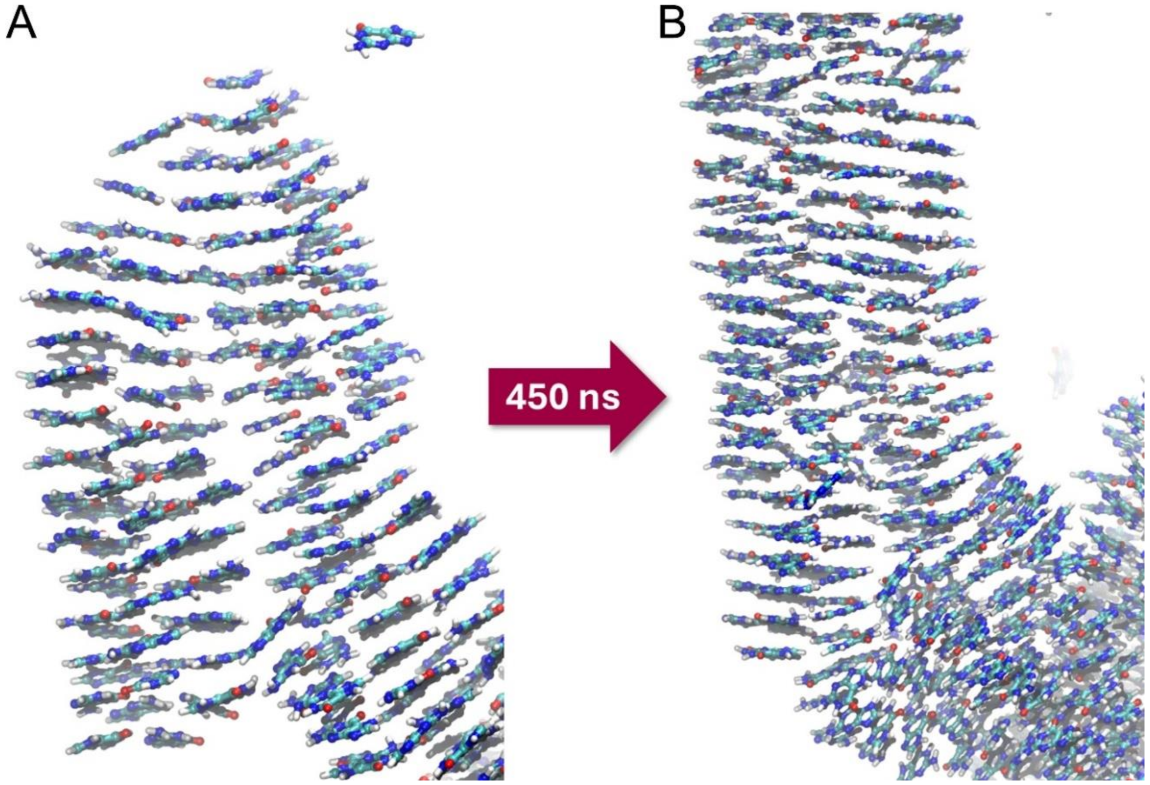

Figure S13. Gradual healing of the domain boundary between two merging guanine helical strands (left) and the progress of converging towards a uniform fiber (without helicity) with the in-plane molecular arrangement of β-guanine 450 ns later (right).

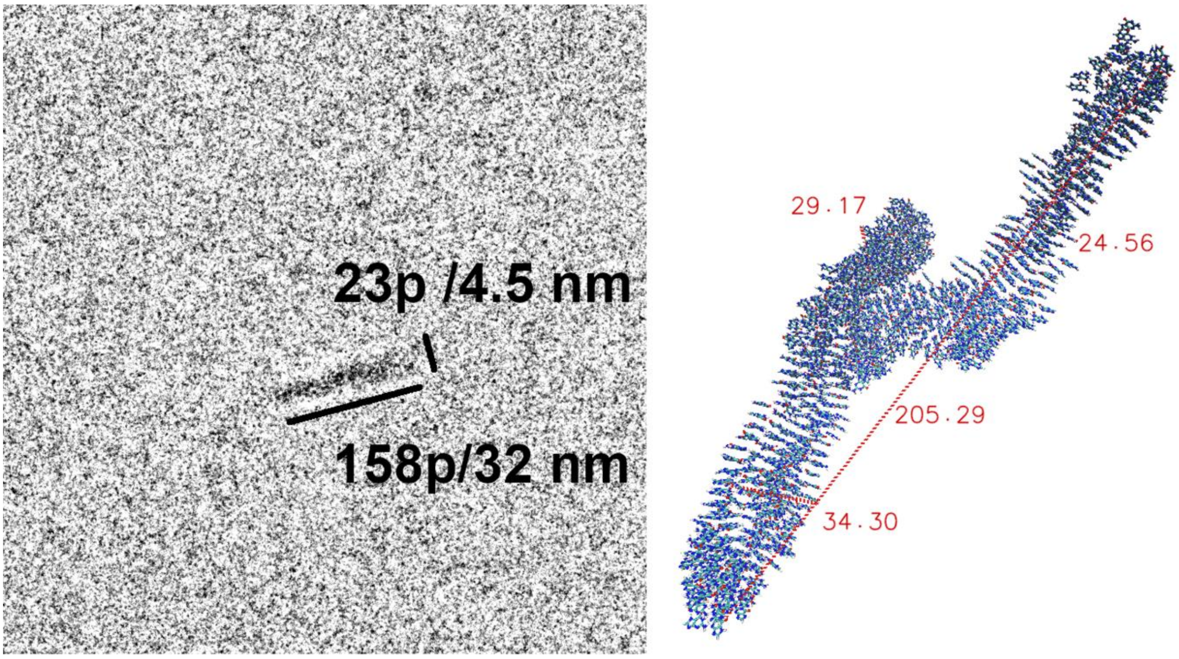

Figure S14. Aspect ratio comparison of guanine fibers: an early-stage fiber from Fig. 2A (left, scale: nm) compared to the largest fiber obtained at the end of the MD simulation – Fig. 5D (right, scale: Å). Both fibers exhibit an approximate aspect ratio of 7:1.

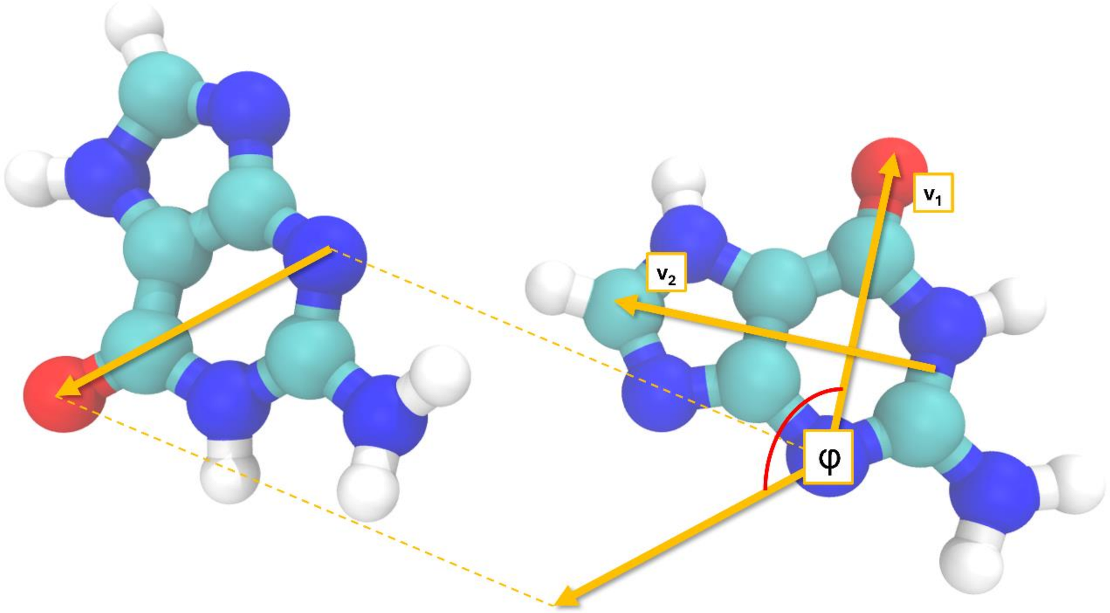

Figure S15. The orientation of a guanine molecule is defined by two vectors v_1 (short axis) and v_2 (long axis). φ is the angle between v_1 of the first molecule and the projection of second molecule's v_1 onto the plane of first molecule. θ is evaluated in the similarly but using v_2.

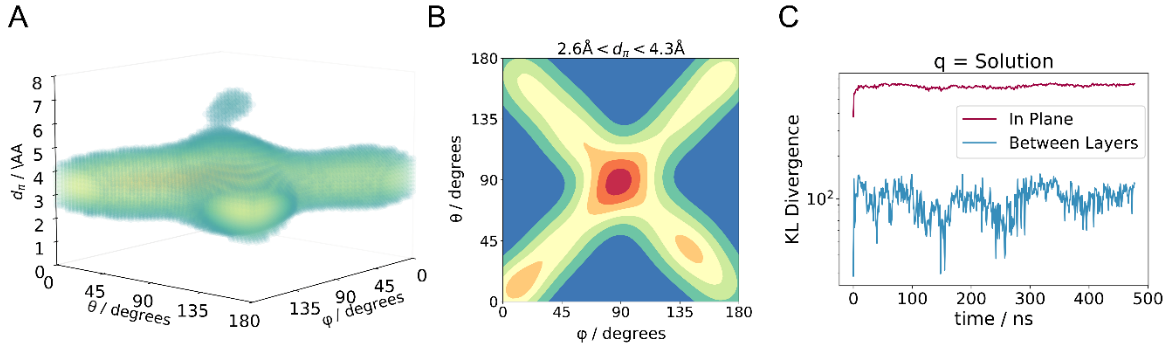

Figure S16. (A) The distribution of characteristic angles φ and θ, resolved along the stacking distance d_π in the supersaturated guanine solution (t=0). **(B)** Cross-section of Fig. 6D corresponding to the preferred relative orientations of molecules in the neighboring layers. **(C)** The KL-divergence as a function of time between the simulated preferred orientation distributions (Fig. 6D) and the corresponding distributions in the supersaturated solution.

S2. Supersaturation calculation

A supersaturation ratio (S) is given by: $S = [A]_x / [A]_{Eq}$

where [A] denotes the concentration (or activity) of species A, and the subscripts x and Eq denote an arbitrary concentration and equilibrium concentration (solubility), respectively.

S3. Supplementary movies

Movie SM1: Timelapse of the 500 ns long simulation of a supersaturated guanine solution in water (Fig. 5A-C). The blue lines indicate the simulation box, and the grey renderings represent the periodic images of the box in two out of the three dimensions.

Movie SM2: Timelapse of the 150 ns long simulation of the fiber grown in Movie SM1 in a two times larger box (Fig. 5D). The blue lines indicate the simulation box.
The resulting fiber obtained at the end of 500 ns of simulation (Movies SM1) was transferred to a larger box (1.5 million atoms). It was equilibrated for further 150 ns to assess its stability in the absence of finite-size effects induced by 3D periodic boundary conditions.

References:

(1) Gur, D.; Pierantoni, M.; Elool Dov, N.; Hirsh, A.; Feldman, Y.; Weiner, S.; Addadi, L. Guanine Crystallization in Aqueous Solutions Enables Control over Crystal Size and Polymorphism. *Cryst. Growth Des.* **2016**, *16* (9), 4975–4980. https://doi.org/10.1021/acs.cgd.6b00566.
(2) Darvishzad, T.; Lubera, T.; Kurek, S. S. Puzzling Aqueous Solubility of Guanine Obscured by the Formation of Nanoparticles. *J. Phys. Chem. B* **2018**, *122* (30), 7497–7502. https://doi.org/10.1021/acs.jpcb.8b04327.
(3) Walrafen, G. E.; Fisher, M. R. [6] Low-Frequency Raman Scattering from Water and Aqueous Solutions: A Direct Measure of Hydrogen Bonding. In *Methods in Enzymology*; Biomembranes Part O: Protons and Water: Structure and Translocation; Academic Press, 1986; Vol. 127, pp 91–105. https://doi.org/10.1016/0076-6879(86)27009-3.
(4) Kresse, G.; Furthmüller, J. Efficient Iterative Schemes for Ab Initio Total-Energy Calculations Using a Plane-Wave Basis Set. *Phys. Rev. B* **1996**, *54* (16), 11169–11186. https://doi.org/10.1103/PhysRevB.54.11169.

(5) Togo, A.; Chaput, L.; Tadano, T.; Tanaka, I. Implementation Strategies in Phonopy and Phono3py. *J. Phys. Condens. Matter* **2023**, *35* (35), 353001. https://doi.org/10.1088/1361-648X/acd831.
(6) Togo, A. First-Principles Phonon Calculations with Phonopy and Phono3py. *J. Phys. Soc. Jpn.* **2023**, *92* (1), 012001. https://doi.org/10.7566/JPSJ.92.012001.
(7) Perdew, J. P.; Burke, K.; Ernzerhof, M. Generalized Gradient Approximation Made Simple. *Phys. Rev. Lett.* **1996**, *77* (18), 3865–3868. https://doi.org/10.1103/PhysRevLett.77.3865.
(8) Grimme, S.; Antony, J.; Ehrlich, S.; Krieg, H. A Consistent and Accurate Ab Initio Parametrization of Density Functional Dispersion Correction (DFT-D) for the 94 Elements H-Pu. *J. Chem. Phys.* **2010**, *132* (15), 154104. https://doi.org/10.1063/1.3382344.
(9) Grimme, S.; Ehrlich, S.; Goerigk, L. Effect of the Damping Function in Dispersion Corrected Density Functional Theory. *J. Comput. Chem.* **2011**, *32* (7), 1456–1465. https://doi.org/10.1002/jcc.21759.
(10) Bedoya-Martínez, N.; Giunchi, A.; Salzillo, T.; Venuti, E.; Della Valle, R. G.; Zojer, E. Toward a Reliable Description of the Lattice Vibrations in Organic Molecular Crystals: The Impact of van Der Waals Interactions. *J. Chem. Theory Comput.* **2018**, *14* (8), 4380–4390. https://doi.org/10.1021/acs.jctc.8b00484.
(11) Giunchi, A.; Pandolfi, L.; Della Valle, R. G.; Salzillo, T.; Venuti, E.; Girlando, A. Lattice Dynamics of Quinacridone Polymorphs: A Combined Raman and Computational Approach. *Cryst. Growth Des.* **2023**, *23* (9), 6765–6773. https://doi.org/10.1021/acs.cgd.3c00634.
(12) Hirsch, A.; Gur, D.; Polishchuk, I.; Levy, D.; Pokroy, B.; Cruz-Cabeza, A. J.; Addadi, L.; Kronik, L.; Leiserowitz, L. "Guanigma": The Revised Structure of Biogenic Anhydrous Guanine. *Chem. Mater.* **2015**, *27* (24), 8289–8297. https://doi.org/10.1021/acs.chemmater.5b03549.
(13) Porezag, D.; Pederson, M. R. Infrared Intensities and Raman-Scattering Activities within Density-Functional Theory. *Phys. Rev. B* **1996**, *54* (11), 7830–7836. https://doi.org/10.1103/PhysRevB.54.7830.
(14) Fonari, A.; Stauffer, S. *Vasp_raman.Py*; 2013.
(15) Abraham, M. J.; Murtola, T.; Schulz, R.; Páll, S.; Smith, J. C.; Hess, B.; Lindahl, E. GROMACS: High Performance Molecular Simulations through Multi-Level Parallelism from Laptops to Supercomputers. *SoftwareX* **2015**, *1–2*, 19–25. https://doi.org/10.1016/j.softx.2015.06.001.
(16) Case, D. A.; Aktulga, H. M.; Belfon, K.; Cerutti, D. S.; Cisneros, G. A.; Cruzeiro, V. W. D.; Forouzesh, N.; Giese, T. J.; Götz, A. W.; Gohlke, H.; Izadi, S.; Kasavajhala, K.; Kaymak, M. C.; King, E.; Kurtzman, T.; Lee, T.-S.; Li, P.; Liu, J.; Luchko, T.; Luo, R.; Manathunga, M.; Machado, M. R.; Nguyen, H. M.; O'Hearn, K. A.; Onufriev, A. V.; Pan, F.; Pantano, S.; Qi, R.; Rahnamoun, A.; Risheh, A.; Schott-Verdugo, S.; Shajan, A.; Swails, J.; Wang, J.; Wei, H.; Wu, X.; Wu, Y.; Zhang, S.; Zhao, S.; Zhu, Q.; Cheatham, T. E. I.; Roe, D. R.; Roitberg, A.; Simmerling, C.; York, D. M.; Nagan, M. C.; Merz, K. M. Jr. AmberTools. *J. Chem. Inf. Model.* **2023**, *63* (20), 6183–6191. https://doi.org/10.1021/acs.jcim.3c01153.
(17) Mark, P.; Nilsson, L. Structure and Dynamics of the TIP3P, SPC, and SPC/E Water Models at 298 K. *J. Phys. Chem. A* **2001**, *105* (43), 9954–9960. https://doi.org/10.1021/jp003020w.
(18) Wagner, A.; Merkelbach, J.; Samperisi, L.; Pinsk, N.; Kariuki, B. M.; Hughes, C. E.; Harris, K. D. M.; Palmer, B. A. Structure Determination of Biogenic Crystals Directly

from 3D Electron Diffraction Data. *Cryst. Growth Des.* **2024**. https://doi.org/10.1021/acs.cgd.3c01290.
(19) Gowers, R. J.; Linke, M.; Barnoud, J.; Reddy, T. J. E.; Melo, M. N.; Seyler, S. L.; Domański, J.; Dotson, D. L.; Buchoux, S.; Kenney, I. M.; Beckstein, O. MDAnalysis: A Python Package for the Rapid Analysis of Molecular Dynamics Simulations. *scipy* **2016**. https://doi.org/10.25080/Majora-629e541a-00e.
(20) Michaud-Agrawal, N.; Denning, E. J.; Woolf, T. B.; Beckstein, O. MDAnalysis: A Toolkit for the Analysis of Molecular Dynamics Simulations. *J. Comput. Chem.* **2011**, *32* (10), 2319–2327. https://doi.org/10.1002/jcc.21787.
(21) Hagberg, A. A.; Schult, D. A.; Swart, P. J. Exploring Network Structure, Dynamics, and Function Using NetworkX. In *Proceedings of the 7th Python in Science Conference*; Varoquaux, G., Vaught, T., Millman, J., Eds.; Pasadena, CA USA, 2008; pp 11–15.
(22) Virtanen, P.; Gommers, R.; Oliphant, T. E.; Haberland, M.; Reddy, T.; Cournapeau, D.; Burovski, E.; Peterson, P.; Weckesser, W.; Bright, J.; van der Walt, S. J.; Brett, M.; Wilson, J.; Millman, K. J.; Mayorov, N.; Nelson, A. R. J.; Jones, E.; Kern, R.; Larson, E.; Carey, C. J.; Polat, İ.; Feng, Y.; Moore, E. W.; VanderPlas, J.; Laxalde, D.; Perktold, J.; Cimrman, R.; Henriksen, I.; Quintero, E. A.; Harris, C. R.; Archibald, A. M.; Ribeiro, A. H.; Pedregosa, F.; van Mulbregt, P. SciPy 1.0: Fundamental Algorithms for Scientific Computing in Python. *Nat. Methods* **2020**, *17* (3), 261–272. https://doi.org/10.1038/s41592-019-0686-2.
(23) Kullback, S.; Leibler, R. A. On Information and Sufficiency. *Ann. Math. Stat.* **1951**, *22* (1), 79–86. https://doi.org/10.1214/aoms/1177729694.
(24) Habraken, W. J. E. M.; Tao, J.; Brylka, L. J.; Friedrich, H.; Bertinetti, L.; Schenk, A. S.; Verch, A.; Dmitrovic, V.; Bomans, P. H. H.; Frederik, P. M.; Laven, J.; van der Schoot, P.; Aichmayer, B.; de With, G.; DeYoreo, J. J.; Sommerdijk, N. A. J. M. Ion-Association Complexes Unite Classical and Non-Classical Theories for the Biomimetic Nucleation of Calcium Phosphate. *Nat. Commun.* **2013**, *4* (1), 1507. https://doi.org/10.1038/ncomms2490.
(25) Turhan, E.; Goldberga, I.; Pötzl, C.; Keil, W.; Guigner, J.-M.; Haßler, M. F. T.; Peterlik, H.; Azaïs, T.; Kurzbach, D. Branched Polymeric Prenucleation Assemblies Initiate Calcium Phosphate Precipitation. *J. Am. Chem. Soc.* **2024**, *146* (37), 25614–25624. https://doi.org/10.1021/jacs.4c07325.
(26) Jmol: an open-source Java viewer for chemical structures in 3D. http://www.jmol.org/
